# How and When May Technostress Impact Workers’ Psycho-Physical Health and Work-Family Interface? A Study during the COVID-19 Pandemic in Italy

**DOI:** 10.3390/ijerph20021266

**Published:** 2023-01-10

**Authors:** Valentina Sommovigo, Chiara Bernuzzi, Georgia Libera Finstad, Ilaria Setti, Paola Gabanelli, Gabriele Giorgi, Elena Fiabane

**Affiliations:** 1 Department of Psychology, Faculty of Medicine and Psychology, Sapienza University of Rome, 00185 Rome, Italy; 2 Department of Brain and Behavioural Sciences, Unit of Applied Psychology, University of Pavia, 27100 Pavia, Italy; 3 Department of Human Sciences, European University of Rome, 00163 Rome, Italy; 4 Istituti Clinici Scientifici Maugeri, IRCCS, Psychology Unit of Pavia Institute, 27100 Pavia, Italy; 5 Istituti Clinici Scientifici Maugeri, IRCCS, Department of Physical and Rehabilitation Medicine, Genova Nervi Institute, 16167 Genova, Italy

**Keywords:** remote working, technostress, COVID-19 pandemic, psycho-physical distress, work-family conflict, fear of COVID-19, workaholism

## Abstract

Although a growing body of research has analyzed the determinants and effects of technostress, it is still unclear how and when technostress would impact workers’ psycho-physical health and work-family interface during the pandemic. To fill this gap, this study tests the mediating mechanisms and the boundary conditions associated with the impact of technostress on workers’ psycho-physical well-being and work-family conflict. A total of 266 Italian workers completed online questionnaires measuring (traditional vs. remote) working modalities, technostress, fear of COVID-19, working excessively, psycho-physical distress, work-family conflict, loss of a loved one due to COVID-19, and resilience. Structural equation models were performed. Results indicated that technostress was positively related to psycho-physical distress and work-family conflict, as mediated by fear of COVID-19 and working excessively, respectively. The loss of a loved one exacerbated the effects of fear of COVID-19 on psycho-physical health, while resilience buffered the effects of working excessively on work-family conflict. Since numerous organizations intend to maintain remote working also after the COVID-19 emergency, it is crucial to study this phenomenon during its peaks of adoption, to prevent its potential negative outcomes. The implications of these findings for theory and practice are discussed.

## 1. Introduction

The new ways of working offered by information and communications technologies (ICTs) have shaped workers’ access to information and people, allowing them to work anywhere, anytime, and from any device [1]. The term remote work defines the situation in which a job is carried out in whole or in part at an alternative worksite than the predefined location [2] and is often used interchangeably with the terms “telework” and “working from home” given the lack of a clear-cut differentiation between concepts (in this study the term remote work will be used). Although the remote work revolution began in the 1980s, the phenomenon has never really spread on a large scale [3]. The COVID-19 pandemic has abruptly changed existing trends, making working from home the norm for millions of workers worldwide [4]. In a very short period of time, the entire economic and social system has had to rely on the use of ICT and the world of work has experienced incalculable pressure to adapt to the use of technologies [5,6]. For instance, European estimates from 2019 show that only one in 20 workers regularly worked remotely, while one third (39%) of EU workers started working from home following the COVID-19 pandemic [3]. Similarly, in Italy (i.e., the European nation with the lowest percentage of remote workers in 2020), the number of remote workers increased by 69% due to the outbreak [7,8]. On the one hand, remote work is associated with various positive outcomes such as enhanced job performance and productivity, greater flexibility and autonomy, more opportunities for collaboration and labor market participation, less commuting time, and lower levels of absenteeism and turnover intentions [9,10,11]. On the other hand, it could lead to detrimental consequences such as social isolation, worse work-life balance, reduction in the sharing of knowledge, creativity and innovation, and new ergonomics and psychosocial risks. In this perspective, technostress (i.e., “the phenomenon of stress experienced by end-users in organizations as a result of their use of ICTs”) is configured as one of the main negative outcomes associated with remote work [2,10,12,13,14] and the increased use of ICT tools during the pandemic due to social distancing measures [15]. Technostress is a multidimensional concept defined for the first time by Brod [16] as the “inability to adapt or cope with new computer technologies in a healthy manner”. From Brod’s first definition, several other models have been proposed (e.g., [14,17,18,19]), outlining a terminological evolution aimed at including all aspects related to digitization. The phenomenon can be operationalized according to different perspectives (i.e., following the stressors or strain conceptualization) and is consensually recognized as a major threat to the well-being of workers, inevitably exposed to new and potentially stressful characteristics of the workplace [20,21]. Despite the call for more research on the topic (e.g., technostress in the context of remote work/hybrid work), there is some evidence that the negative effects of technostress involve manifestations such as headaches, cardiovascular problems, mood changes, fatigue, anxiety, emotional exhaustion, concentration problems, work-family conflict, decreased levels of productivity, satisfaction and commitment, burnout, absenteeism, and higher turnover levels [22,23,24,25,26]. It should be noted that ICTs per se are not stressful, but rather certain characteristics related to their use (e.g., intrusiveness, unreliability, complexity) [13,27]. These same characteristics are part of the main models and psychometric tools used to evaluate the phenomenon. For the purposes of this study, we will follow the transactional model of Ragu-Nathan et al. [14] analyzing technostress as techno-overload (i.e., increased workload and short deadlines related to ICT), techno-invasion (i.e., constant connectivity due to ICT and invasion of the personal sphere) and techno-complexity (i.e., the intrinsic complexity of new technologies and the consequent feeling of inadequacy). As many companies intend to maintain a hybrid way of working (i.e., unlike remote working where employees work from a location away from the traditional workspace where the employer is based, hybrid workers work partly in the workspace and partly remotely) even after the COVID-19 emergency [8], more research is needed to unravel how companies can maximize the benefits of remote work and minimize the risks to employees’ mental health. Nevertheless, only a few studies have focused on how technostress may affect employees’ mental health [28], with previous research on this topic focusing primarily on its work-related effects [13].

### 1.1. Technostress and Psycho-Physical Distress

In the pandemic context, information overload due to the increased use of ICT (i.e., techno-overload), the presence of more blurred boundaries between work and family domains due to the constant connectivity allowed by new technologies (i.e., techno-invasion), and the difficulty in learning how to utilize digital tools (i.e., techno-complexity) were all factors contributing to increased psycho-physical distress levels among workers [8]. Accordingly, previous studies have identified technostress as a predictor of psychological disorders, such as anxiety, mental fatigue, depression, and sleeping problems [29,30], and physical symptoms, such as gastrointestinal problems, high blood pressure, and insomnia [30,31]. There is also empirical evidence that intensive use of ICT and resulting technostress can lead users to produce higher levels of stress hormones (i.e., adrenaline, cortisol, and alpha amylase), and augmented activity of the cardiovascular system [32,33]. Hence, we hypothesize:

 **Hypothesis 1.**  *Technostress will be positively associated with psycho-physical distress.*

Although the use of ICTs has increased exponentially exposing workers to serious risks to their well-being, little is known about how and when technostress may be associated with psycho-physical distress (i.e., the changes in the psychophysical state of an individual, with emphasis on the presence of distress symptoms, difficulties in social performing and coping with problems, together with loss of self-confidence) in the context of the COVID-19 pandemic. As for the underlying mechanisms, one of the phenomena that has spread in what has been called the “psychological pandemic” is a state of fear associated with COVID-19 [34,35]. The feeling of fear is one of the most common responses to highly traumatic events, such as epidemics [36]. Especially in the case of COVID-19, there are numerous factors that can elicit this type of response such as fear of contagion, fear of spreading the virus, prolonged isolation, fear of not receiving adequate treatment, insecurity, and loss, just to name a few. Despite the potentially adaptive role in promoting safe behaviors (e.g., respecting hygiene and distancing rules) fear of COVID-19 is actually associated with lower levels of psychological health [37,38]. For example, a recent meta-analysis with a total sample of 70,407 individuals showed that fear of COVID-19 was strongly related to mental health issues such as anxiety, traumatic stress, distress, and depression [39].

Investigating whether fear of COVID-19 may be an explanatory mechanism for this relationship seems to be particularly relevant because the literature from past [40] and current outbreaks showed that the spread of a pandemic results in a sharp increase in fear and worries related to the virus, which (when excessive) may exacerbate the damage of the disease itself with negative effects at the individual and societal levels [41]. Indeed, to date, numerous studies have found a positive link between stress and fear of COVID-19, suggesting that the stronger the perceived stress, the greater the fear of the virus [42]. There is also evidence to support the idea that fear of COVID-19 can act as a mediator, such as between stress and job satisfaction [42] or between stress-related uncertainty and well-being [43]. However, only a few studies have analyzed the association between technostress and fear (or anxiety) of COVID-19, consistently finding a positive relationship between these two [44,45]. One possible explanation for this positive association could lie in the relationship between cognitive and emotional functions and chronic stress. Chronic work-related stress can affect workers’ cognitive structure and profoundly influence their sense-making process. Specifically, workers experiencing stressful situations may have a reduced ability to down-regulate emotions and may not be able to process negative contextual stimuli, which are perceived as more threatening [46]. For example, the results of the study conducted by Golkar et al. [47] in individuals suffering from chronic work-related stress highlighted an alteration in functional couplings within the emotion- and stress-processing limbic networks associated with a reduced ability to regulate negative emotions. At the neurophysiological level, Rosenkranz et al. [48] demonstrated that chronic stress increases fear and the amygdala’s lateral nuclei neural excitability. Chronic stress may thus lead to an overactive fear/anxiety circuit, impairing at the same time the activity of other areas (e.g., hippocampus and medial prefrontal cortex) aimed at fear inhibition [49]. Following the Cognitive Vulnerability Model of the etiology of fear, after a fear-relevant stimulus has triggered a vulnerability schema, two parallel processes occur. An automatic affective reaction and a slower cognitive appraisal (similar to Lazarus and Folkman’s primary and secondary appraisal) [50]. Technostress may impact both processes, impairing the interpretation of COVID-19-related stimuli. Similarly, following the transactional model of stress, emotions reflect how an individual views the environment under certain circumstances [48]. Ongoing cognitive appraisals are an intrinsic part of the emotion, as the quantity and quality of emotions (e.g., fear, anger, anxiety) depend on a particular pattern of appraisal and factors related to the cognitive activity (e.g., perception, thoughts). For example, secondary appraisal (i.e., the process of evaluating coping resources) plays a central role in shaping the subsequent emotional outcome. Technostress can then be appraised as a hindrance stressor that triggers negative emotions because it is a condition that hinders one’s personal growth and goal attainment, and for which future losses are possible [51]. Thus, according to the transactional model [52], negative emotions are likely to result from harm and threats to valued outcomes. Such anticipation of potential harm or failure can burden a worker psychologically and hinder his/her coping ability to manage such a situation, leading to negative emotions [53] and various types of job strain, including physical symptoms [54,55]. In this sense, experiencing high levels of technostress could influence fear-related circuits while leading the individual to interpret environmental stimuli, including those related to the pandemic, in a more negative way and these could be more likely to elicit a fear response. If workers are techno-stressed, they could assess their resources as not sufficient to face the hypothetical environmental threats related to the pandemic, developing more fears. Technostress is demanding both emotionally and cognitively, depleting the individual’s resources. Hence, following the perspective on the cognitive consequences of stress and the transactional model and the neurophysiological paradigm, technostress may influence workers’ emotional circuits and the interpretation of the events, making them more susceptible to interpreting COVID-19-related aspects as threatening, thus leading to higher levels of fear. The pandemic has modified workers’ traditional work modes and required the massive use of ICTs at the expense of face-to-face interactions, leaving them potentially isolated from their work community and at increased risk of becoming techno-stressed [8]. This, in turn, could arouse more fears of the unknown virus [49] as the factors underlying the fear of COVID-19 (e.g., contagion, loss, isolation) could be perceived as more threatening. Thus, there is evidence that fear of COVID-19 can severely affect mental well-being [56], exacerbate pre-existing mental health complications, and elicit stress reactions [57,58]. Hence, we propose:

 **Hypothesis 2.**  *Technostress will be positively related to fear of COVID-19 (a) which, in turn, will be positively associated with psycho-physical distress (b).*

Another unclear factor concerns when this process occurs. The topic of death and mortality is generally associated with feelings of apprehension and anxiety capable of influencing human behaviors and mental processes [50]. According to the Terror Management Theory (TMT), in response to the topic of death individuals use both proximal and distal defenses aimed at reducing the discomfort associated with it [58,59]. When faced with death salience, these defenses are activated by what is defined as the anxiety buffer system to protect the individual from psycho-physical distress [59]. However, the relationship between death anxiety and psycho-physical distress appears to be exacerbated during the pandemic and individuals seem to be more at risk of developing psychological symptoms. Available data suggest that the actual loss of a family member or friend due to the direct effect of SARS-CoV-2 infection profoundly affects mental health [60,61]. In this respect, since the loss of a loved one due to the virus has been identified as one of the most stressful events during the COVID-19 outbreak that may trigger detrimental effects on employees’ health [62], it is reasonable to expect that such a loss would exacerbate the impact of fear of COVID-19 on employees’ psycho-physical distress [63]. Hence, we propose:

 **Hypothesis 3.**  *The loss of a loved one due to COVID-19 will moderate the positive relationship between technostress and psycho-physical distress through fear of COVID-19 so that this relationship will be stronger when employees suffer from the loss of a loved one.*

### 1.2. Technostress and Work-Family Conflict

Grounded in role stress and inter-role conflict theories [64], work-family conflict is described as a form of inter-role conflict that occurs when the role pressures from the work and family domains are mutually incompatible [65]. Indeed, the boundaries between work and non-work are blurred so that people’s behavior and emotional states from one domain may spill over into another [66]. The construct of work-family conflict is composed of three components: the behavior-based conflict (i.e., the behaviors required by the work role are incompatible with those required for the family role), the strain-based conflict (i.e., the work-related strain negatively affects an individual’s performance in the family domain), and the time-based conflict (i.e., the amount of time needed by the work role prevents the fulfillment of the family role’s expectations; [67]). To date, research (e.g., [68,69]) has shown a positive association between technostress and work-family conflict. Thus, when employees have to deploy time and energy to learn and handle new technologies at work, the resultant techno-overload and techno-complexity may leave them with less time and energy to devote to their family responsibilities [70]. Similarly, dealing with technology-related pressures and technology-assisted supplemental work can make workers too tired and overwhelmed to fully participate in their family life and effectively fulfill the expectations of their family members (e.g., [70,71]). Moreover, when workers feel frustrated because of the resource depletion related to high levels of techno-overload, they can bring home these negative emotional states [72]. Furthermore, although the use of ICTs to perform work tasks may lead to greater flexibility that may help employees better combine work and family life, this may also make them feel connected and performative 24 h a day [73]. Constant accessibility can create the expectation of having to respond immediately and be indiscriminately accessible, implicitly suggesting not to detach from work [74]. As a result, the boundaries between work and private life are more permeable, and workers may perceive a greater invasion of technology into family life, finding it difficult to disconnect from work [8]. This techno-invasion can deter them from giving the proper attention to their family, making it difficult to fulfill their family roles successfully [70], resulting in work-family conflict [75]. Hence, we hypothesize the following:

 **Hypothesis 4.**  *Technostress will be positively related to work-family conflict.*

Most prior studies have focused on only time- (e.g., inflexible work schedule, shift work) and strain-based (e.g., role conflict, boundary-spanning activities) sources of work-family conflict, while less attention has been paid to behavior-based sources (e.g., expectations for secretiveness and objectivity; [65,76]). In this study, we focus on the behavioral component of workaholism (i.e., a form of work addiction that leads to investing an excessive amount of time and energy into work; [68,69]), namely working excessively as a behavior-based source of work-family conflict. Thus, the tendency to work excessively hard and beyond what is expected to satisfy one’s job demands is likely to take place at the expense of the family role [77]. To date, the association between the use of ICTs and workaholism has been mainly studied in relation to the phenomenon of techno-addiction (i.e., an uncontrollable compulsion to use ICT “everywhere and anytime” and to excessively utilize it for long periods together with anxiety when not utilizing it; [19]) or to the fact that workaholic tendencies could lead individuals to use smartphone devices [78] or ICTs [79]. Nevertheless, recently scholars have recognized the interest in studying whether technostress might increase the risk of workaholism, calling for more research on this topic [80]. The underlying idea is that the anxiety stemming from the use of new technologies for work may facilitate the occurrence of workaholism. This is because situational factors, including the work context, can exacerbate workaholic behavior among workers predisposed to developing this compulsive behavior [81]. Precisely, a recent study has revealed that in individualistic European societies the impact of the COVID-19 pandemic, combined with the economic crisis and unemployment risk, translated primarily into working excessively [82]. This behavioral component of workaholism is of particular interest for the study of the work-life interface because, unlike its cognitive component (i.e., working compulsively), it is strongly related to work-family conflict [83,84]. Indeed, when working excessively, workers devote a substantial amount of time and energy to their professional activity, which leaves them with scarce resources left to devote to their family life [83]. This behavioral tendency can interfere with the performance of a worker’s family role and generate fatigue states that make it difficult to satisfy the demands of the family role [83]. To date, although workaholism was found to be an antecedent of technostress among remote workers during the outbreak, the call to investigate the presence of an inverse relationship between these two constructs has not yet been addressed [78, 80]. Thus, it is still unknown whether and when technostress would lead employees to work excessively and then experience work-family conflict. To fill this gap, this study aims to examine whether technostress would lead employees to work excessively, and then experience work-family conflict. Hence, we propose:

 **Hypothesis 5.**  *Technostress will be positively related to tendencies to work excessively (a) which, in turn, will be positively associated with work-family conflict (b).*

To date, it is still unknown under which personal boundary conditions technostress would drive employees who work excessively to experience work-family conflict. In this regard, resilience, which represents a personal resource that helps in handling stressful situations, may play a protective role against work-family conflict [85], even when a person works excessively. In fact, highly resilient people can have rich pools of resources to draw from to sustain their efforts to work excessive hours while maintaining, at the same time, enough energy to meet family needs [86], at least in the short term. However, to date, there is no empirical evidence to support this. To fill this gap, we hypothesize:

 **Hypothesis 6.**  *Resilience will moderate the positive relationship between technostress and work-family conflict through tendencies to work excessively such that this relationship will be weaker when employees have high levels of resilience.*

Overall, this study had two main purposes: (a) to analyze whether the relationship between technostress and psycho-physical distress would be mediated by fear of COVID-19 and conditional on the loss of a loved one due to COVID-19; (b) to examine whether technostress would be associated with work-family conflict, both directly and indirectly, as mediated by tendencies to work excessively and conditionally on resilience levels.

**Figure 1:**
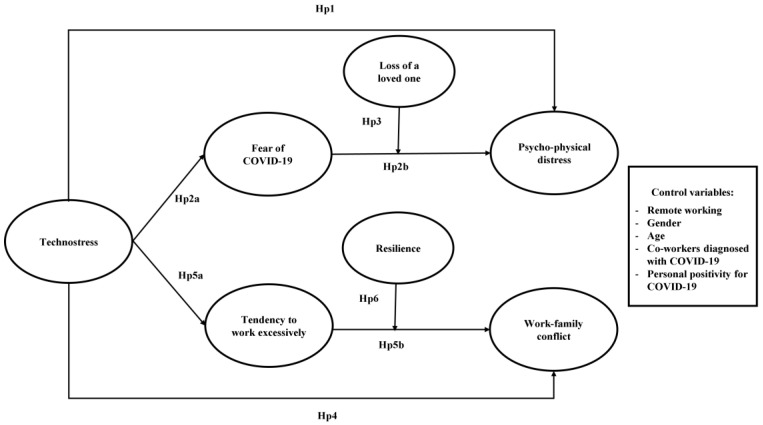
Graphical illustration of the hypothesized relationships.

By pursuing these goals, this study contributes to the existing literature on technostress in several ways. First, responding to a call for more research on the effects of technostress on employees’ mental health [75], this study sheds light on how and when technostress may lead employees to develop psycho-physical distress during the COVID-19 pandemic. Second, by studying the tendency to work excessively as an underlying mechanism linking technostress with work-family conflict, this study responds to a call for more research on the link between technostress and workaholism [8]. Third, by analyzing whether this relationship could be conditional on resilience levels, this study provides practical insights into how to prevent work-family conflict during health emergencies. Figure 1 shows our conceptual model.

## 2. Materials and Methods

### 2.1. Participants and Procedure

This cross-sectional research was conducted during the COVID-19 emergency (January–March 2021) in observance of the Declaration of Helsinki. After obtaining ethical approval from the Research Ethics Committee of the University of Pavia, the survey was administered online using a form from a spreadsheet in Google Sheets. The link to submit the survey was distributed through social network sites (i.e., LinkedIn, Instagram, Facebook, Twitter, and WhatsApp). The inclusion criteria were as follows: to be at least 18 years of age, to be working at the time of filing, and to provide an informed consent form. The questionnaire cover sheet informed the respondents about the study objectives and ensured both the voluntary nature of their participation and the anonymity of the responses. The resulting convenience sample was composed of 287 workers. We excluded 21 respondents because of incomplete responses (i.e., less than 60% of the survey). Thus, the final sample included 266 Italian workers. Most of the respondents were women (62.60%) with an average age of 39.40 years (*SD* = 12.26) who were employed mainly as highly specialized professionals (40.90%) in Northern Italy (52.60%). More than half of the participants (56.80%) had begun to work remotely due to COVID-19. All participants reported that their use of ICTs for work was considerably increased during the pandemic period (100.00%). Most of the respondents were tested for COVID-19 (60.50%), received a negative diagnosis (91.00%), and had some of their loved ones who were working in healthcare settings (54.5%) and had been diagnosed with COVID-19 (56.80%), without being among fatalities (86.40%). Slightly less than half of them (46.4%) had colleagues who were positive for COVID-19.

### 2.2. Measurements

Resilience was assessed using the ten-item Connor-Davidson Resilience Scale in its Italian version [87]. Participants indicated how much they agreed with each assertion about ways of responding to stressful situations on a five-point Likert scale (0 = almost always false, 4 = almost always true).

Working excessively was measured using five items from the Italian version of the Dutch Work Addiction Scale [88]. Respondents reported how frequently they tended to allocate an exceptional amount of time to their work on a four-point Likert scale (1 = never or almost never, 4 = almost always or always).

Technostress was evaluated using the eleven-item Italian Technostress Creators Scale [8], which includes three dimensions: techno-overload, techno-invasion, and techno-complexity. Respondents reported how frequently they experienced technostress situations on a five-point Likert scale (1 = strongly disagree, 5 = strongly agree). This measurement has shown good factorial validity among remote and traditional workers, and invariance of the measurement structure across these two groups [8].

Work-family conflict was evaluated using five items from the Work-Family Conflict scale [89]. Participants indicated how much they agreed with each statement describing work-to-family conflict situations on a seven-point Likert scale (1 = completely disagree, 7 = completely agree).

Psycho-physical distress was measured using the twelve-item General Health Questionnaire in its Italian version [90], which includes three subscales: social dysfunction, general dysphoria, and loss of confidence. Participants indicated whether and how their psycho-physical health differed from their usual state on a four-point Likert scale (positively worded items: 0 = better than usual, 3 = much less than usual; negatively worded items: 0 = not at all, 3 = much more than usual).

Fear of COVID-19 was measured using the seven-item Italian version of the Fear of COVID-19 Scale [91]. Respondents indicated how much they agreed with each statement on fear and worries related to the virus on a five-point Likert scale (0 = strongly disagree, 5 = strongly agree).

The loss of a loved one due to COVID-19 was evaluated using a single dichotomous item (0 = no, 1 = yes; i.e., have any of your loved ones been among the fatalities?).

Covariates. We controlled for gender (0 = male, 1 = female), age (in years), and remote work during the COVID-19 pandemic (i.e., currently, are you working remotely because of COVID-19?). The latter was measured using a dichotomous item (0 = no, 1 = yes). Furthermore, participants completed a checklist on their experience with COVID-19, which included dichotomous items (0 = no, 1 = yes), analyzing: being tested for COVID-19 (i.e., Have you been tested for COVID-19?), personal (i.e., Have you been diagnosed with COVID-19?) and co-worker positivity for COVID-19 (i.e., Have any of your colleagues been diagnosed with COVID-19?), having family or friends who were diagnosed with the virus (i.e., Have any of your loved ones been diagnosed with COVID-19?) and working in healthcare settings (i.e., Are any of your loved ones working in health care settings?).

## 3. Results

### 3.1. Descriptive Analyses

Firstly, to verify the suitability of our sample size, we conducted a power analysis for a multiple regression analysis having ten predictors using the program G*Power [92]. The results of this power analysis, which was computed using an alpha of 0.05, a power of 0.95, and a medium effect size, revealed that a sample of at least 172 participants was necessary, indicating that our sample size was acceptable. The data were screened for outliers, multicollinearity, normality, and correlations were explored using SPSS 25 ([93] see Table 1). All the correlations among the study constructs were in the expected direction. Remote working, gender, age, and personal and colleague positivity for COVID-19 were used as control variables due to their statistically significant correlations with the study constructs.

The missing data ranged from a low of 0% for the items on the resilience scale to a high of 1.5% for the items on the general health scale. The results of Little’s MCAR test were not statistically significant (χ^2^ = 843.19, *p* = 0.06), indicating that the data were completely missing at random.

### 3.2. Independent T-Test Analyses and Analyses of Variance

The results of the independent t-test analysis (see Supplemental Tables S1 and S2) showed that employees who began to work remotely due to COVID-19 reported higher levels of technostress (M = 2.57, SD = 0.86) and fear of COVID-19 (M = 2.41, SD = 1.08) than those who did not (technostress: M = 2.11, SD = 0.88; fear: M = 2.04, SD = 0.94). Statistically significant differences were found between men and women such that women reported higher levels of fear of COVID-19 (M = 2.38, SD = 1.04), work excessively tendencies (M = 2.92, SD = 0.66), psycho-physical distress (M = 3.64, SD = 1.66), and work-family conflict (M = 3.64, SD = 1.66) as well as lower levels of resilience (M = 2.85, SD = 0.54) than men (fear: M = 1.89, SD = 0.91; work excessively: M = 2.71, SD = 0.77; psycho-physical distress: M = 0.95, SD = 0.62; work-family conflict: M = 3.23, SD = 1.48; resilience: M = 3.08, SD = 0.49). However, no statistically significant differences in technostress levels were found across gender. Workers who were positive for COVID-19 (M = 4.17, SD = 1.85) were more likely to experience work-family conflict than those who were not (M = 3.43, SD = 1.57). Workers who had positive co-workers reported higher levels of technostress (M = 2.44, SD = 0.96) than those who had not (M = 2.19, SD = 0.84). Workers who suffered from the loss of a loved one due to COVID-19 reported higher levels of fear of COVID-19 (M = 2.88, SD = 1.33) and work-family conflict (M = 4.11, SD = 1.78) than those who did not (fear: M = 2.09, SD = 0.92; work-family conflict: M = 3.39, SD = 1.56).

The results of analyses of variance (ANOVAs) indicated that groups with different years of age differed from each other in levels of technostress, fear of COVID-19, tendencies of working excessively, and psycho-physical malaise. Bonferroni posthoc comparisons showed that workers over 50 years old reported higher levels of technostress (M = 2.59, SD = 0.78) and fear of COVID-19 (M = 2.61, SD = 1.02) than workers under 30 years of age (technostress: M = 1.90, SD = 0.73, fear: M = 1.95, SD = 0.96). Workers aged between 31 and 40 reported higher tendencies to work excessively (M = 2.38, SD = 1.04) than those over 50 years old (M = 2.38, SD = 1.04) and higher levels of psycho-physical distress (M = 2.38, SD = 1.04) in comparison with those aged between 41 and 50 (M = 2.38, SD = 1.04).

### 3.3. Confirmatory Factor Analyses and Assessment of Common Method Bias

The results of the confirmatory factor analysis (see Table 2), which was performed with the Full Information Maximum Likelihood method using Mplus version 8, indicated that the six-factor model outperformed any alternative model (χ^2^ [1160] = 2411.32, RMSEA = 0.06, SRMR = 0.07, CFI = 0.89, TLI = 0.88), supporting the distinctiveness of the study variables. However, to improve the fit indices of this model (χ^2^ [1149] = 1970.06, RMSEA = 0.05, SRMR = 0.07, CFI = 0.90, TLI = 0.90), we correlated the errors of six pairs of items on the technostress scale, two pairs of items on the fear of COVID-19 scale, and three pairs of items on the psycho-physical health scale due to the high intercorrelations existing between them. The subsequent models were built considering the modification indices that were used in this satisfactory model.

The results of Harman’s single-factor test indicated that the first factor explained only 21.06% of the variance. Additionally, the hypothesized six-factor model yielded a better fit to the data after the inclusion of the unmeasured latent method factor. Moreover, the unmeasured latent method factor explained 24.00% of the total variance (which is less than 25%, the average amount of method variance observed in self-report research [94]), suggesting that common method variance was unlikely to be a major concern in this study.

### 3.4. Hypotheses Testing

We conducted a mediation model using bootstrapping analyses and a bias-corrected 95% confidence interval with a resampling procedure of 1000 bootstrap samples (χ^2^ [948] = 1693.08, RMSEA = 0.05, SRMR = 0.07, CFI = 0.90, TLI = 0.90). In this model (see Table 3), technostress was positively related to psycho-physical distress (β = 0.26, SE = 0.08, *p* < 0.01, 95% CI [0.10, 0.41]), fear of COVID-19 (β = 0.27, SE = 0.07, *p* < 0.001, 95% CI [0.13, 0.44]), working excessively (β = 0.43, SE = 0.07, *p* < 0.001, 95% CI [0.13, 0.64]), and work-family conflict (β = 0.36, SE = 0.06, *p* < 0.001, 95% CI [0.23, 0.50]), while controlling for remote work, gender, age, personal and colleague positivity for COVID-19. Fear of COVID-19 was, in turn, positively associated with psycho-physical distress (β = 0.38, SE = 0.07, *p* < 0.05, 95% CI [0.24, 0.56]) and partially mediated the relationship between technostress and psycho-physical distress (β = 0.09, SE = 0.03, *p* < 0.05, 95% CI [0.02, 0.16]). Working excessively was, in turn, positively related to work-family conflict (β = 0.45, SE = 0.07, *p* < 0.001, 95% CI [0.31, 0.58]) and partially mediated the association between technostress and work-family conflict (β = 0.17, SE = 0.04, *p* < 0.05, 95% CI [0.10, 0.28]). The mediation effects of fear of COVID-19 and working excessively remained independent of the addition or not of covariates (mediation model without covariates: χ^2^ [726] = 1443.86, RMSEA = 0.06, SRMR = 0.08, CFI = 0.90, TLI = 0.90). Overall, technostress was related to psycho-physical distress and work-family conflict, directly and indirectly, as mediated by fear of COVID-19 and working excessively, respectively. Therefore, Hypotheses 1, 2a, 2b, 4, 5a, and 5b were confirmed.

**Figure 2:**
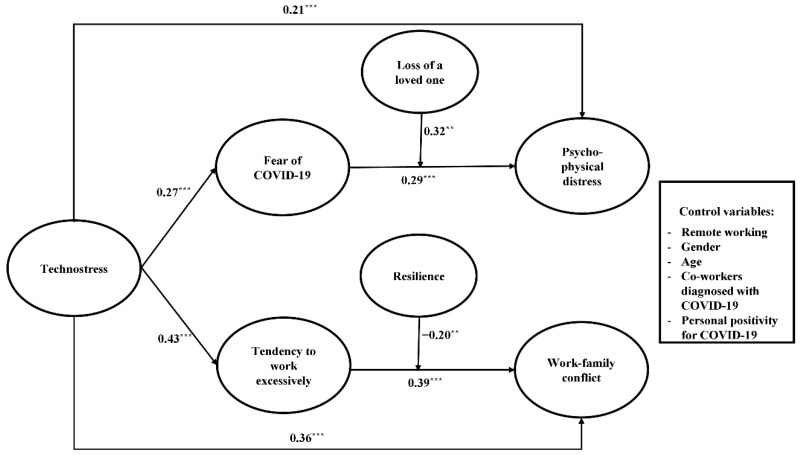
Path coefficients of the hypothesized model. ** *p* < 0.01; *** *p* < 0.001.

**Figure 3:**
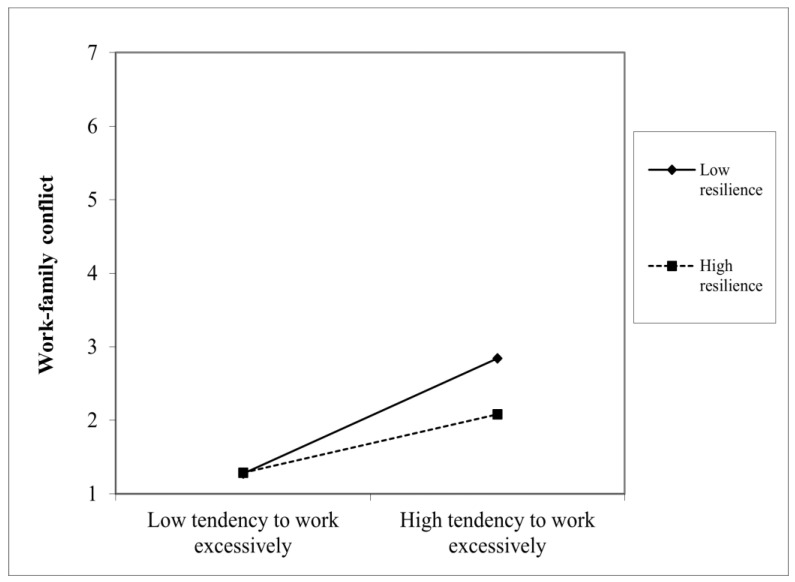
Moderating effects of resilience on the relationship between working excessively and work-family conflict.

Next, different moderated mediation models were conducted. The model with the loss of a loved one due to COVID-19 and resilience as moderators was the best fitting model (i.e., lowest AIC and BIC values; AIC = 35,237.99, BIC = 35,900.27) compared to those that included socio-demographic variables (e.g., age: AIC = 74,702.14, BIC = 74,777.25) or other COVID-19-related variables (e.g., having loved ones working in healthcare settings: AIC = 35,796.23, BIC = 36,854.25) as moderators. The relationship between fear of COVID-19 and psycho-physical distress was exacerbated when workers had lost a loved one due to COVID-19 (β = 0.32, SE = 0.12, *p* < 0.01; see Table 4 and Figure 2). Indeed, the moderated mediation effect was statistically significant only for workers who had lost a loved one (β = 0.08, SE = 0.02, *p* < 0.01). Moreover, the association between working excessively and work-family conflict was buffered by resilience (β = −0.20, SE = 0.07, *p* < 0.01), such that workers with low (β = 0.46, SE = *0*.11, *p* < 0.001) or moderate (β = 0.31, SE = 0.08, *p* < 0.001) resilience levels were susceptible to the consequences of working excessively in terms of work-family conflict, while those with high resilience levels did not experience work-family conflict (β = 0.15, SE = 0.09, ns), even when they tended to work excessively. Examination of the interaction plot (see Figure 3) showed that highly resilient workers reported much lower increases in work-family conflict than those who scored low on resilience when passing from low to high tendencies to work excessively. Thus, Hypotheses 3 and 6 were supported.

## 4. Discussion

This study tries to clarify how and when technostress may impact workers’ psycho-physical health and work-life interface during the COVID-19 pandemic in Italy. Employees who had begun to work remotely due to COVID-19 were more likely to be techno-stressed than traditional workers. These findings further support the association between remote work and technostress [78,80] as well as the differential psychological impact of the pandemic experienced by frontline and remote workers [95]. Technostress was positively related to psycho-physical distress, directly and indirectly, as mediated by fear of COVID-19. Thus, techno-stressed individuals were more afraid of COVID-19. As hypothesized, one possible explanation is that employees suffering from technostress may have an altered perception of the environment, perceiving external pandemic-related stimuli as more concerning. Furthermore, workers may assess their own resources as insufficient to cope with possible threats, thus developing more fears. Another possible explanation is that increased use of ICTs raises specific concerns (e.g., fear of losing one’s job due to ICTs) and hinders the creation of meaningful psychological connections with work communities [45]. This may result in an additional burden for many employees, making them more vulnerable to experiencing COVID-19-related anxiety [45] and fear. While moderate levels of fear could drive individuals to adopt safety behaviors and be more responsible, high levels of fear (especially over an extended period) can threaten individuals’ mental health [41]. Precisely, this study showed that the uncertain threat posed by the virus evoked fear among techno-stressed workers, seriously compromising their psychological health [41]. However, this mediated effect was statistically significant for workers who had lost a loved one only. As suggested by previous research, the loss of a loved one is associated with the onset of psychological symptoms [96]. In addition, recent evidence highlight how grief related to losing a significant other because of SARS-CoV-2 seems to have a greater impact than deaths from natural causes [60]. Bereaved families of COVID-19 victims might go through psychological crises that further exacerbate the impact of COVID-19 fear on their psycho-physical health [97]. In fact, the sudden death of a loved one is often accompanied by fear of the future because it may threaten individuals’ family stability and financial conditions, in addition to generating complications in social interactions and stigmatization [97]. These results add to a growing body of literature on the negative effects of technostress [13,26] by identifying fear of COVID-19 as an underlying mechanism linking technostress to workers’ psycho-physical distress and loss of a loved one as a boundary condition under which this may happen.

Technostress was positively related to work-family conflict, directly and indirectly, as mediated by the tendency to work excessively. As demonstrated in previous studies [68,69], technostress may spill over into the family domain because of the “always-on” culture and the nature of work-related ICT arrangements that create an unbridgeable gap between how an individual is expected to behave during family time and job requests as mediated through ICTs (e.g., mobile email). Thus, when work technology seriously permeates family life, individuals may experience work-family conflict because they have less time and energy to fulfill their duties in the family domain. Moreover, our findings indicated that this relationship could be mediated by working excessively and moderated by resilience. On the one hand, this suggests that techno-stressors may represent work-related factors that may trigger workaholic behaviors probably due to the absence of clearly defined boundaries between the domains, as well as the lack of recreation opportunities due to COVID-19-related restrictions [98]. On the other hand, this provides further support for the protective role of resilience against work-family conflict [66]. Indeed, resilient workers are well-equipped to sustain their efforts to work excessively, while, in meantime, fulfilling their family obligations. By identifying working excessively as an underlying mechanism that explains the relationship between technostress and work-family conflict, this study answers the call for more research on the link between technostress and workaholism [8].

Our group comparisons based on (traditional vs. remote) work modalities indicated that remote workers were more likely to experience higher levels of technostress and fear of COVID-19 than those working onsite. During the pandemic, many Italian workers were forced to use ICTs for remote working for the first time, frequently without first receiving any type of remote work training [99], making them particularly vulnerable to experiencing technostress [8]. Thus, since remote working involves spatial and temporal separation from other collaborators, remote workers were likely to spend more time in virtual communication and collaboration using digital technologies than onsite workers [100,101], which might have made them more vulnerable to technostress. Given the barriers generated by physical separation, remote workers involved in virtual communication via ICTs were more likely to experience difficulties in exchanging and interpreting implicit knowledge [102], identifying who knew what, and comprehending where to locate knowledge and expertise [103]. Moreover, although during the COVID-19 pandemic, the physical distance protected workers from contracting the virus, the social isolation experienced was accompanied by worries and uncertainties related to the virus [104]. Thus, remote workers could be more afraid of the virus because of a lack of contact with reality and continuous exposure to frightening news about the virus through social media, which could have feed their fear of the virus [104].

The results of our group comparisons based on gender indicated that women were more likely to experience work-family conflict than men. This may be due to gender inequalities in the distribution of household chores and childcare due to traditional gender-role expectations (e.g., [105]). This model still persists within Italian society which has traditionally been characterized by a substantial gender gap in the labor market and the family [106]. This condition has been exacerbated by the outbreak that further increased gender inequalities, placing the burden of childcare and housework duties mainly on women [107]. Thus, women had to work and, meanwhile, perform time-consuming activities associated with family life, such as taking care of their children and elderly family members [107]. Indeed, during the pandemic, Italian women spent, respectively, 26% and 10% more time on household chores and childcare than men [106]. Given that schools in Italy were closed longer than in any other European country [108], many women, while working from home, had to care for their children with no or limited access to caregiver resources [109]. Women were also mainly responsible for following their children’s education through home-schooling or remote learning offered by their school during school closures and reduced academic schedules due to the COVID-19 pandemic [109]. Then, since the burden of extra-work related to childcare and housework fell disproportionately on women, it is not surprising that, especially during the pandemic, women had more difficulties in reconciling conflicting requests from work and home domains and managing different roles than men [110]. This may also contribute to the higher psycho-physical distress levels reported by women in comparison with men. These different distress conditions can also reflect the gender-related differences in lifestyles and the socio-economic status during the pandemic [111]. In this view, women might be more distressed as they were more frequently overwhelmed by unpaid extra-work (e.g., childcare, household chores [109]), and at greater risk of job losses and income reductions than men [112]. An alternative explanation might be related to the lower levels of resilience reported by women in our sample compared with men. Less resilient women may be more likely to experience psycho-physical distress and work-family conflict, as they are less equipped against resource loss [66]. Moreover, women reported higher levels of fear of COVID-19 than men. This result is not surprising as numerous studies have found that women felt afraid of and anxious about COVID-19 more frequently than men in many countries, such as Japan [109], and the United States [111]. Thus, women tend to feel more intense affective states and experience negative emotions, including fear, more frequently [111].

Additionally, according to the literature [113], our results indicated that technostress increased with age such that older workers were more vulnerable to developing it. This may be due to age-related physical degeneration of processes relevant to technology use (e.g., hearing, vision, and fine motor skills [114]) and to generational differences in digital literacy between younger (i.e., digital natives) and older workers (i.e., digital immigrants [115]). Older workers also reported higher levels of fear of COVID-19 probably because they perceived themselves to be at particular risk of developing the adverse effects of COVID-19-related infections, such as hospitalization, intensive care, need for ventilators to breathe, mortality, and more serious sequelae following the infection than other age groups in the population [116,117,118]. Workers aged between 31 and 40 reported higher tendencies to work excessively and higher levels of psycho-physical distress than other age groups. Middle-aged workers could tend to work more excessively than other age groups as they may be more concerned about their career development, their income, and the economic security of their families [82], experiencing greater psycho-physical distress.

Finally, the results of group comparisons based on COVID-19 variables showed that workers who were positive for COVID-19 reported higher levels of work-family conflict than those who were not. A possible explanation may be that the presence of concerns about the risk of infecting family members and the need to respect social-distancing restrictions depleted the energy of these workers, interfering with their ability to fulfill family requests [66]. Moreover, workers who lost a loved one reported higher levels of fear of COVID-19 and work-family conflict than those who did not. This may be because bereaved workers could gain a first-hand experience of the dangerousness of the virus and go through a severe psychological crisis, together with concerns about their family stability and financial condition [108]. Furthermore, workers who had positive co-workers reported higher levels of technostress than those who did not probably because they had to cover absent colleagues, making their techno-overload heavier [97].

### Limitations and Future Research Directions

Despite the precautions, this study has some limitations that should be addressed. This cross-sectional study relied only on online self-report measures. Thus, future studies should adopt a longitudinal design and collect data from multiple sources, trying to reach non-Internet users. Furthermore, our findings are limited to the Italian working population during the pandemic. In line with prior studies (e.g., [119]), our sample was composed of Italian remote and traditional workers who used ICTs during the pandemic period. This is because the literature indicated that technostress had increased among traditional and remote workers during the pandemic because all workers were forced to start using and learning new digital technologies and social media applications as their main tools of communication and collaboration due to social-distancing and containment measurements posed by the pandemic [120]. However, given that remote workers reported higher levels of technostress than traditional workers and are generally more vulnerable to technostress, we controlled our results for (traditional vs. remote) work modalities, in line with what was done by prior studies (e.g., [8]). More research is needed to clarify how and when technostress can produce negative consequences among remote workers. For instance, future studies could analyze whether employees who always work remotely from home can differentiate themselves in technostress experiences from employees who work partially remotely, alternating days of work at home, and days of work in the workplace. Replications on larger and more nationally representative and gender-balanced samples of Italian workers in non-pandemic times are needed to increase the generalizability of the findings provided by the current research. In addition, we analyzed the relationship between technostress and fear of COVID-19, but other psychosocial, physical, and social factors capable of eliciting a distress response could be investigated in the future. Likewise, this study focused on one behavior-based source of work-family conflict only, namely working excessively. However, other behavior-, time-, and strain-based sources might contribute to explaining through which mechanisms technostress could be related to work-family conflict. Then, more research is needed to identify a more comprehensive range of mediating factors and (personal and situational) boundary conditions to reach a better understanding of how and when technostress is linked to work-family conflict. Indeed, the extent to which technostress can generate work-family conflict and psycho-physical distress can be expected to vary depending upon numerous factors, such as family/household size and conditions (e.g., age-school children), the organizational position, the degree of perceived job (in-)security, cultural dimensions, and gender role expectations, which can be investigated in future research.

Future studies should also investigate the role of personality traits that were found to be precursors of workaholism (e.g., neuroticism) [121]). More research is also needed to identify other personal and environmental resources (e.g., professional self-efficacy; managerial support) [122]) that could buffer the detrimental effects of technostress on workers’ well-being. Moreover, selection bias cannot be ruled out because of the non-probability convenience sampling strategy. However, several studies used social networks to collect data during the COVID-19 pandemic due to the restrictions on social interactions that were mandated in several European countries [123]. Nevertheless, given the “healthy worker effect” [124,125], it might be that our research participants were healthy enough to complete the survey, whereas those who experienced more technostress or were more distressed might have not had the energy to participate in this study due to their poor health conditions [125]. Future studies could consider including an incentive for respondents to encourage broader participation to reduce this bias [124].

This research suggests implications for practice. Since our findings indicated that technostress may negatively affect workers’ well-being and work-family interface, organizations could provide their staff (especially remote and older workers) with on-demand training programs on email and time management to adequately prepare them to use ICTs. Informative events on how to prevent ICT use-related risks could also be useful to prevent technostress, in addition to the implementation of a technical support service for assistance with digital tools. Older workers (who were found to be more likely to develop technostress) could also benefit from reverse mentoring in the use of ICTs and techno-effectiveness training. Additionally, given that techno creators were found to be conducive to work-family conflict, organizations could consider introducing family-friendly practices relating to disconnection during non-work times, such as clear corporate guidelines about email response times, to reduce techno-invasion. To reduce the tendency to work excessively which was found to be a mediator of the relationship between technostress and work-family conflict, organizations could provide group training on cognitive-behavioral stress management and relaxation techniques, especially to women workers. Organizations could consider introducing a reward system that encourages appropriate behaviors rather than inappropriate, excessive work behavior. Since resilience was found to be a protective factor in work-family conflict, workers (especially women) will benefit from psychological resilience training. Moreover, since technostress can engender workers’ well-being, organizations could introduce a psychological support service to support workers, especially those with low resilience and those who lost a loved one due to COVID-19. Moreover, to reduce fear of COVID-19 that is conducive to psycho-physical distress, organizations should take care of employees’ worries about the virus by providing them with clear information about the procedures implemented by the organization to contain the spread of the virus and available personal protective equipment, preventing the dissemination of contradictory messages that would further exacerbate their fears. The lessons learned about the effects of technostress during the COVID-19 pandemic could help organizations better manage remote work in the future.

## 5. Conclusions

This study moves an important step forward in the technostress literature, as it is one of the first investigations to provide an explicative model that investigates through which psychological mechanisms and under which boundary conditions technostress is linked to psycho-physical distress and work-family conflict in the pandemic context. In doing so, this is the first study to identify fear of COVID-19 as a mechanism explaining how technostress is related to psycho-physical distress and the loss of a loved one due to COVID-19 as a factor exacerbating this relationship, adding to the literature on outbreaks. Additionally, this is the first study to prove the mediating role of the tendency to work excessively in the relationship between technostress and work-family conflict, enriching the array of behavior-based sources of work-family conflict and answering calls for more research on the technostress-workaholism relationship. Finally, by identifying resilience as a buffer of this relationship, this study provides further evidence for the protective role of this personal resource against work-family conflict. We hope these findings will encourage future attempts to clarify when and how technostress can affect workers’ well-being and work-family interface, which is crucial due to the ever-increasing importance of digital tools in today’s workplaces.

## Figures and Tables

**Table 1:** Intercorrelations and descriptive statistics among study variables (*n* = 266).

	M	SD	Skewness	Kurtosis	1	2	3	4	5	6	7	8	9	10	11	12	13	14
1. Remote working	-	-	-	-	-													
2. Technostress	2.31	0.90	0.52	−0.46	0.25 **	**0.88**												
3. Fear of COVID-19	2.10	1.02	0.85	0.01	0.18 **	0.28 *	**0.91**											
4. Working excessively	2.83	0.71	−0.34	−0.45	0.05	0.30 **	0.16 **	**0.72**										
5. Psycho-physical distress	1.14	0.67	0.71	−0.06	0.10	0.27 **	0.42 **	0.20 **	**0.89**									
6. Work-family conflict	3.49	1.61	0.23	−0.82	0.09	0.50 **	0.27 **	0.46 **	0.35 **	**0.89**								
7. Resilience	2.93	0.53	−0.34	−0.11	−0.05	0.01	−0.08	0.12 *	−0.35 **	−0.03	**0.80**							
8. Gender	-	-	-	-	0.14 *	0.05	0.23 **	0.15 *	0.21 **	0.12 *	−0.21 **	-						
9. Age	39.40	12.26	-	-	0.17 *	0.26 **	0.18 **	−0.08	−0.11	0.06	0.03	0.13 *	-					
10. Being tested for COVID-19	-	-	-	-	−0.06	−0.01	−0.07	0.07	−0.01	0.06	−0.04	0.09	−0.03	-				
11. Personal positivity	-	-	-	-	−0.09	0.08	0.01	0.01	0.02	0.13 *	0.11	0.03	−0.12	0.25 **	-			
12. Colleague’s positivity	-	-	-	-	0.13 *	0.14 *	0.08	−0.06	0.07	0.07	0.01	0.07	0.10	0.10	0.13 *	-		
13. Family/friends’ positivity	-	-	-	-	0.09	0.09	0.09	0.08	0.09	0.08	0.04	0.08	−0.06	0.08	0.19 **	0.25 **	-	
14. Loss of a loved one	-	-	-	-	−0.03	0.06	0.26 **	0.08	0.06	0.15	0.10	0.12 *	0.10	−0.06	0.10	0.20 **	0.18 **	-
15. Family working in healthcare	-	-	-	-	0.01	−0.01	0.04	0.07	0.07	0.11	0.07	0.03	0.03	0.17 **	0.13 *	0.15 *	0.29 **	0.03

Boldfaced numbers on the diagonal represent Cronbach’s alpha; M = means; SD = standard deviations. * *p* < 0.05; ** *p* < 0.01.

**Table 2:** Fit indices for the six-factor model and the alternative models (*n* = 266).

Model	χ^2^	df	*p*	RMSEA	90% RMSEA	SRMR	CFI	TLI
Six-factor model_meth ^n^	1781.860	1099	0.00	0.04	[0.04, 0.05]	0.05	0.90	0.90
Six-factor model_mo ^m^	1970.061	1149	0.00	0.05	[0.04, 0.06]	0.07	0.90	0.90
Six-factor model ^l^	2411.325	1160	0.00	0.06	[0.06, 0.07]	0.07	0.89	0.88
Five-factor model2 ^i^	2538.719	1165	0.00	0.07	[0.06, 0.07]	0.07	0.78	0.77
Five-factor model1 ^h^	3067.571	1165	0.00	0.08	[0.07, 0.08]	0.09	0.69	0.68
Four-factor model2 ^g^	3241.569	1169	0.00	0.08	[0.07, 0.08]	0.09	0.67	0.65
Four-factor model1 ^f^	3284.148	1169	0.00	0.08	[0.08, 0.09]	0.10	0.66	0.64
Three-factor model2 ^e^	4138.958	1172	0.00	0.09	[0.09, 0.10]	0.12	0.52	0.50
Three-factor model1 ^d^	4154.705	1172	0.00	0.10	[0.09, 0.10]	0.12	0.52	0.50
Two-factor model 2 ^c^	4561.390	1174	0.00	0.10	[0.10, 0.11]	0.13	0.45	0.43
Two-factor model 1 ^b^	4668.066	1174	0.00	0.11	[0.10, 0.11]	0.12	0.44	0.42
One-factor model ^a^	5148.710	1175	0.00	0.11	[0.10, 0.12]	0.13	0.36	0.33

df = degree of freedom; RMSEA = Root Mean Square Error of Approximation; SRMR = Standardized Root Mean Square Residuals; CFI = Comparative Fit Index; TLI = Tucker-Lewis Index. ^a^ All indicators load on a single factor. ^b^ Technostress, working excessively, fear, WFC, and psycho-physical distress load on one factor; resilience loads on a second factor. ^c^ Technostress, working excessively, and fear load on one factor; resilience, WFC, and distress load on a second factor. ^d^ Technostress, working excessively, and fear load on the first factor; WFC, and distress load on the second factor; resilience loads on the third factor. ^e^ Technostress, working excessively load on the first factor; WFC, distress load on the second factor; resilience loads on the third factor; fear loads on the fourth factor. ^f^ Technostress loads on the first factor; fear and distress load on the second factor; working excessively, WFC load on the third factor; resilience loads on the fourth factor. ^g^ Technostress loads on the first factor; fear and distress load on the second factor; WFC, working excessively load on the third factor; resilience loads on the fourth factor. ^h^ Technostress loads on the first factor; fear loads on the second factor; distress, WFC load on the third factor; working excessively loads on the fourth factor; resilience loads on the fifth factor. ^i^ Technostress loads on the first factor; fear loads on the second factor; distress loads on the third factor; WFC, working excessively load on the fourth factor; resilience loads on the fifth factor. ^l^ Technostress fear, distress, WFC, working excessively, and resilience load on their respective factor. ^m^ Prior model allowing correlations for six pairs of items from the technostress scale (item 4 with 1, item 3 with item 1, item 2 with item 1, item 2 with item 1, item 4 with item 1, item 3 with item 4), for two pairs of items from the fear scale (item 6 with item 3, item 5 with item 2) and three pairs of items from distress (item 11 with 10, item 4 with item 3, item 5 with item 2) due to the high intercorrelations existing between them. ^n^ Previous model with the inclusion of a common method latent variable on which making all the items load.

**Table 3:** Fit indices and standardized direct and indirect effects for the mediation model.

Model (Outcome)	χ^2^	df	*p*	RMSEA	SRMR	CFI	TLI
Model 1	1693.076	948	0.000	0.05 [0.05,0.06]	0.07	0.90	0.90
Standardized direct and indirect effects							
Effects—Model 1			Estimate		S.E.	95% CI	
Remote working → Technostress			0.23 **		0.07	[0.09,0.39]	
Age → Technostress			0.22 **		0.07	[0.09,0.36]	
Colleagues positive for COVID-19 → Technostress			0.06		0.07	[−0.07,0.19]	
Age → Working excessively			−0.21 **		0.07	[−0.36, −0.11]	
Gender → Working excessively			0.17 *		0.08	[0.05,0.27]	
Working excessively → Work-family conflict			0.45 ***		0.07	[0.31,0.58]	
Gender → Work-family conflict			0.02		0.05	[−0.09,0.13]	
COVID-19 positivity → Work-family conflict			0.08		0.05	[−0.01,0.18]	
Technostress → Work-family conflict			0.36 ***		0.07	[0.23,0.50]	
Technostress → Working excessively			0.43 ***		0.07	[0.13,0.64]	
Technostress → Working excessively → Work-family conflict			0.17 *		0.04	[0.10,0.28]	
Total effects on work-family conflict			0.56 ***		0.06	[0.44,0.68]	
Remote working → Fear of COVID-19			0.08		0.06	[−0.03,0.20]	
Gender → Fear of COVID-19			0.17 **		0.06	[0.05,0.29]	
Loss of loved ones → Psycho-physical distress			0.59 ***		0.19	[0.36,0.66]	
Ag e→ Fear of COVID-19			0.07		0.06	[−0.05,0.19]	
Fear of COVID-19 → Psycho-physical distress			0.38 ***		0.07	[0.24,0.56]	
Gender → Psycho-physical distress			0.16 **		0.06	[0.05,0.27]	
Age → Psycho-physical distress			−0.23 ***		0.06	[−0.36, −0.11]	
Technostress → Psycho-physical distress			0.26 **		0.08	[0.10,0.41]	
Technostress → Fear of COVID-19			0.27 *		0.07	[0.13,0.44]	
Technostress → Fear of COVID-19 → Psycho-physical distress			0.09 **		0.03	[0.02,0.16]	
Total effects on psycho-physical distress			0.35 ***		0.08	[0.19,0.51]	

df = degree of freedom; RMSEA = Root Mean Square Error of Approximation; SRMR = Standardized Root Mean Square Residuals; CFI = Comparative Fit Index; TLI = Tucker-Lewis Index. * *p* < 0.05; ** *p* < 0.01; *** *p* < 0.001.

**Table 4:** Path coefficients and conditional effects for the moderated mediation model.

Paths	Effects	
	Estimate	S.E.
Remote working→ Technostress	0.23 ***	0.06
Age→ Technostress	0.22 ***	0.06
Colleagues’ positivity for COVID-19→ Technostress	0.06	0.06
Age→ Working excessively	−0.21 **	0.07
Gender→ Working excessively	0.12 *	0.05
Technostress→ Working excessively	0.43 ***	0.07
Working excessively → Work-family conflict	0.39 ***	0.07
Gender→ Work-family conflict	−0.01	0.05
Personal positivity for COVID-19→ Work-family conflict	0.10	0.05
Gender→ Resilience	−0.15 *	0.02
Resilience→ Work-family conflict	−0.11	0.09
Working excessively × Resilience→ Work-family conflict	−0.20 **	0.07
Technostress→ Work-family conflict	0.36 ***	0.06
Remote working→ Fear of COVID-19	0.06	0.06
Gender→ Fear of COVID-19	0.20 **	0.06
Age→ Fear of COVID-19	0.08	0.06
Technostress→ Fear of COVID-19	0.27 ***	0.07
Fear of COVID-19→ Psycho-physical distress	0.29 ***	0.07
Fear of COVID-19 × Loss→ Psycho-physical distress	0.32 **	0.12
Loss→ Psycho-physical distress	0.59 **	0.19
Gender→ Psycho-physical distress	0.16 **	0.05
Age→ Psycho-physical distress	−0.20 ***	0.06
Techno-distress→ Psycho-physical distress	0.21 ***	0.06
Technostress→ Working excessively × Low Resilience →Work-family conflict	0.46 ***	0.11
Technostress→ Working excessively × Moderate Resilience → Work-family conflict	0.31 ***	0.08
Technostress→ Working excessively × High Resilience→ Work-family conflict	0.15	0.09
Total effects for Low Resilience	1.12 ***	0.07
Total effects for Moderate Resilience	0.97 ***	0.13
Total effects for High Resilience	0.81 ***	0.13
Technostress→ Fear of COVID-19 × No loss of a loved one→ Psycho-physical distress	−0.04	0.02
Technostress→ Fear of COVID-19 × Loss of a loved one→ Psycho-physical distress	0.08 **	0.02
Total effects for no loss of a loved one	0.11 ***	0.04
Total effects for loss of a loved one	0.20 ***	0.04

* *p* < 0.05; ** *p* < 0.01; *** *p* < 0.001. S.E. = standard errors.

## Data Availability

The data that support the findings of this study are available from the corresponding author upon reasonable request.

## Supplementary Materials

The following supporting information can be downloaded at: https://www.mdpi.com/article/10.3390/ijerph20021266/s1, Table S1: Independent t-test analyses among groups differing in work mode (in presence vs. remote working) and gender with reference to the study’s variables; Table S2: Independent t-test analyses among groups differing in COVID-19-related experience variables with reference to the study’s variables; Table S3: ANOVAs across age groups with reference to the study’s variables.
