# Supplementary material for: How and When May Technostress Impact Workers’ Psycho-Physical Health and Work-Family Interface? A Study during the COVID-19 Pandemic in Italy
Source: Int J Environ Res Public Health. 2023 Jan 10;20(2):1266. doi: 10.3390/ijerph20021266 (PMC9859582; doi:10.3390/ijerph20021266)
Supplement: Supplementary file 1 [file ijerph-20-01266-s001.zip › ijerph-2095059-supplementary.pdf]

Supplemental Table S1. Independent t-test analyses among groups differing in work mode (in presence vs. remote working) and gender with reference to the study's variables.

	On worksite (n=151)		Remote working (n=115)		t	*p*	95% IC		Cohen's *d*
	M	DS	M	DS			LI	LS	
Techno distress	2.11	.88	2.57	.86	-4.23	.000	-.67	-.24	.53
Fear of COVID	2.04	.94	2.41	1.08	-2.95	.003	-.61	-.12	.36
Work excessively	2.81	.75	2.88	.66	-.82	.41	-.24	.10	-
GHQ	1.08	.65	1.22	.69	-1.66	.099	-.30	.03	-
WFC	3.36	1.67	3.66	1.51	-1.51	.132	-.69	.09	-
Resilience	2.95	.52	2.90	.54	.74	.459	-.08	.18	-
	Male (n=99)		Female (n=166)		t	*p*	95% CI		Cohen's *d*
	M	DS	M	DS			LI	LS	
Techno distress	2.25	.87	2.34	.92	-.81	.420	-.32	.13	-
Fear of COVID	1.89	.91	2.38	1.04	-3.89	.000	-.74	-.24	.50
Work excessively	2.71	.77	2.92	.66	-2.42	.016	-.39	-.04	.29
GHQ	.95	.62	1.25	.68	-3.54	.000	-.46	-.13	.46
WFC	3.23	1.48	3.64	1.66	-2.03	.044	-.81	.01	.26
Resilience	3.08	.49	2.85	.54	3.44	.001	.10	.35	.45

Supplemental Table S2. Independent t-test analyses among groups differing in COVID-19-related experience variables with reference to the study's variables.

	No COVID (n=242)		COVID positivity (n=24)		t	*p*	95% CI		Cohen's *d*
	M	DS	M	DS			LI	LS	
Techno distress	2.28	.86	2.52	1.23	-1.25	.210	-.63	.14	-
Fear of COVID	2.20	.99	2.23	1.26	-.13	.895	-.46	.40	-
Work excessively	2.84	.70	2.86	.87	-.14	.888	-.32	.28	-
GHQ	1.13	.65	1.19	.89	-.35	.725	-.33	.23	-
WFC	3.43	1.57	4.17	1.85	-2.17	.031	-.91	-.07	.43
Resilience	2.91	.52	3.12	.59	-1.87	.063	-.43	.01	-
	No loss (n=229)		Loss of loved ones (n=36)		t	*p*	95% CI		Cohen's *d*
	M	DS	M	DS			LI	LS	
Techno distress	2.28	.87	2.43	1.08	-.93	.354	-.09	.25	-
Fear of COVID	2.09	.92	2.88	1.33	-4.44	.000	-.78	.18	.69
Work excessively	2.82	.70	2.98	.78	-1.26	.207	-.41	.09	-
GHQ	1.12	.65	1.25	.78	-1.03	.306	-.36	.11	-
WFC	3.39	1.56	4.11	1.78	-2.53	.012	-.98	-.16	.43
Resilience	2.91	.54	3.06	.43	-1.62	.106	-.34	.03	-
	No positive coworkers (n=142)		Positive coworkers (n=123)		t	*p*	95% CI		Cohen's *d*
	M	DS	M	DS			LI	LS	
Techno distress	2.19	.84	2.44	.96	-2.27	.024	-.47	-.03	.28
Fear of COVID	2.13	.84	2.29	1.20	-1.25	.213	-.40	.09	-
Work excessively	2.88	.69	2.79	.74	.91	.364	-.09	.25	-
GHQ	1.10	.63	1.19	.72	-1.10	.272	-.25	.07	-
WFC	3.39	1.55	3.61	1.68	-1.14	.256	-.62	.16	-
Resilience	2.92	.55	2.94	.51	-.23	.815	-.14	.11	-

Supplemental Table S3. ANOVAs across age groups with reference to the study's variables.

Variable	Age	*M*	*SD*	*F*	*95% IC*	
					LL	*UL*
Techno distress	<30 years old	1.90	.73	9.46^{***}	1.75	2.06
	31-40 years old	2.37	.98		2.14	2.60
	41-50 years old	2.44	.94		2.14	2.75
	>50 years old	2.59	.78		2.40	2.77
Fear of COVID	<30 years old	1.95	.96	5.58^{**}	1.74	2.15
	31-40 years old	2.21	1.07		1.96	2.46
	41-50 years old	2.09	.88		1.80	2.37
	>50 years old	2.61	1.02		2.35	2.86
Work excessively	<30 years old	2.75	.67	3.04^{*}	2.61	2.89
	31-40 years old	3.03	.77		2.85	3.21
	41-50 years old	2.88	.76		2.63	3.12
	>50 years old	2.71	.61		2.56	2.86
GHQ	<30 years old	1.15	.68	2.85^{*}	1.00	1.29
	31-40 years old	1.31	.75		1.13	1.49
	41-50 years old	.94	.58		.75	1.13
	>50 years old	1.09	.58		.94	1.23
WFC	<30 years old	3.17	1.62	2.40	2.82	2.82
	31-40 years old	3.77	1.87		3.33	3.35
	41-50 years old	3.23	1.36		2.79	2.79
	>50 years old	3.61	1.27		3.30	3.30
Resilience	<30 years old	2.88	.50	.14	2.77	2.30
	31-40 years old	2.94	.59		2.80	3.08
	41-50 years old	2.88	.51		2.71	3.05
	>50 years old	2.98	.49		2.85	3.10

Note. M= mean; DS= standard deviation; 95% CI= 95% confidence intervals; LL=lower limit; UL=upper limit.
